# Anxiety and support in breast cancer: is this different for affluent and deprived women? A questionnaire study

**DOI:** 10.1038/sj.bjc.6602072

**Published:** 2004-07-27

**Authors:** U Macleod, S Ross, L Fallowfield, G C M Watt

**Affiliations:** 1 General Practice and Primary Care, Division of Community Health Sciences, University of Glasgow, 1 Horselethill Road, Glasgow G12 9LX, UK; 2 Maternal, Infant and Reproductive Health Research Unit, Centre for Research in Women's Health, Sunnybrook and Women's College Health Sciences Centre, 790 Bay Street, Toronto, Ontario, Canada M8V 2Y4; 3 Psychosocial Oncology Group Cancer Research UK, Brighton and Sussex Medical School, University of Sussex, Falmer, Brighton BN1 9QG, UK

**Keywords:** breast cancer, socioeconomic status, SF-36, psychosocial

## Abstract

A postal questionnaire was sent to affluent and deprived women with breast cancer in order to compare psychosocial aspects of care with the purpose of understanding the balance of care and explaining why deprived women have poorer outcomes. Data were collected regarding reported sources of information, SF-36 scores and ongoing causes of anxiety. The results demonstrate that affluent women were more likely to have received information from their hospital specialist (94.8 *vs* 76.0%) and from a breast care nurse (70.1 *vs* 40.0%) than deprived women. They were also more likely to have received information from magazines (50.6 *vs* 33.0%), newspapers (45.5 *vs* 22.0%) and television news (45.5 *vs* 26.0%). Deprived women had poorer SF-36 scores than affluent women, and reported greater anxiety about money (12.2 *vs* 2.8%), other health problems (22.1 *vs* 8.2%) and family problems (17.5 *vs* 6.9%). Personal and professional support is clearly important for patients with breast cancer. Health professionals need to be aware of the greater psychological distress demonstrated by deprived women, even some years after diagnosis with breast cancer, and seek to address it.

There is a higher incidence of breast cancer in affluent women than in socioeconomically deprived women (Tomatis, 1990). However, several studies have shown deprived women to have poorer survival from breast cancer. Previous work carried out by the authors (Macleod *et al*, 2000a) confirmed earlier research (Carnon *et al*, 1994; Schrijvers *et al*, 1995) in showing no relationship between deprivation and pathological prognostic factors in women with early breast cancer. In addition, we have shown (Macleod *et al*, 2000b) that the NHS delivered health care equitably to women with breast cancer who lived in affluent and deprived areas. In particular, socioeconomic status did not affect breast surgery, radiotherapy or adjuvant treatments.

The care of women with breast cancer is not, however, limited to surgical and oncological treatments but also includes provision of information and psychosocial care and support. Although breast cancer is largely treated in hospitals by specialist surgeons and oncologists (Gillis and Hole, 1996) women spend the majority of their time after diagnosis in the community. Our study has shown that there were greater numbers of consultations with general practitioners following diagnosis than before diagnosis for all women, but that the greatest number were for women living in deprived areas (Macleod *et al*, 2000b).

The relationship between breast cancer outcomes and psychosocial factors is complex and several studies have shown a link between socioeconomic status and broader psychosocial issues. Social class has been shown to have an effect on psychiatric outcome at 12 months after mastectomy with women in lower social classes having the worst outcome (Dean, 1987). Other research has suggested that women with breast cancer from lower socioeconomic groups may be particularly receptive to interventions that will enhance their quality of life (McEvoy and McCorkle, 1990). Any exploration of care for women with breast cancer must therefore include an investigation into the psychosocial aspects of care.

In order to identify sources of information and psychosocial support available, investigate how these may differ between affluent and deprived women, and develop a more complete picture of the balance of care for breast cancer in different socioeconomic groups, we carried out a questionnaire survey asking women directly about these aspects of care.

## METHOD

A postal questionnaire was sent to all women participating in a larger study investigating general practice and hospital care for breast cancer (Macleod *et al*, 2000b). The general practitioners of the participants were contacted in order to ensure that no new circumstances had arisen which would make it undesirable for the women to receive a questionnaire. The women were between 3 and 5 years following diagnosis with early (i.e., operable) breast cancer. The questionnaire included a measure of general health status and psychological well being (SF-36, Ware *et al*, 1993) and questions on information sources and help-seeking behaviour. The respondents were asked where they had received information about breast cancer: from family and friends, GP, hospital specialist, breast care nurse, alternative practitioner, three voluntary organisations who have offices in Glasgow, (CANCERBACUP, Breast Cancer Care, Tak Tent), magazines, newspapers, books, leaflets, or television. They were asked if they had obtained information from other sources, and if so to state these, and say which of the sources had been most helpful. A separate question asked, where respondents had received advice on practical problems, and gave them the same list of options.

In order to investigate help-seeking behaviour, women were asked what action they were likely to take if they became anxious about their breast problem. The following options were presented: keep it to yourself, speak to family or friends, speak to your GP, contact breast care nurse, contact hospital specialist, contact voluntary organisation, such as CANCERBACUP or Tak Tent and respondents were asked to reply *yes/no/possibly*. They were then asked which of these had been most helpful in the past. The purpose of this question was to ask about behaviour related to the diagnosis of breast cancer and in so doing discover whether the women perceived their GP to have a role in this. In addition to the psychological questions asked in SF-36, the questionnaire asked: ‘Do you worry about any of the following: money problems, job security, breast cancer, other health problems, family problems and relationship problems?’ The respondents were asked to grade their responses *very much/somewhat/a little/not at all.*

## RESULTS

### Information issues

Women were most likely to have obtained information about breast cancer from their hospital specialist, but women living in affluent areas were more likely to have done so than women living in deprived areas (94.8 *vs* 76.0%, *P*=0.0007, Table 1

**Table 1:** Information-seeking behaviour of women living in affluent and deprived areas from individuals, health care workers and voluntary organisations

**Information source**	**Affluent *n* (% of affluent group) Total *n*=77**	**Deprived *n* (% of deprived group) Total *n*=100**	***χ*^2^ test results**
Family and friends	23 (29.9%)	16 (16.0%)	*χ*^2^=4.87, DF=1
			*P*=0.027
GP	44 (57.1%)	54 (54.0%)	*χ*^2^=0.17, DF=1
			*P*=0.68
Hospital specialist	73 (94.8%)	76 (76.0%)	*χ*^2^=11.55, DF=1
			*P*=0.0007
Breast care nurse	54 (70.1%)	40 (40.0%)	*χ*^2^=15.86, DF=1
			*P*=0.00007
Alternative practitioner	2 (2.6%)	2 (2.0%)	Fisher's exact test
			*P*=0.79
			
*Voluntary organisations*			
CANCERBACUP	12 (15.8%)	7 (7.0%)	*χ*^2^=3.46, DF=1
			*P*=0.06
Breast cancer care	18 (23.4%)	11 (11.0%)	*χ*^2^=4.86, DF=1
			*P*=0.03
Tak Tent	4 (5.2%)	1 (1.0%)	Fisher's exact test
			*P*=0.09

This table shows those who answered yes to the statement: ‘We would like to know about where you have obtained *information* relating to your breast problem (e.g., causes, treatment)’.

). Women from affluent areas were also more likely to have obtained information from breast care nurses (70.1 *vs* 40.0%, *P*=0.00007), and to have acquired information from their family and friends (29.9 *vs* 16.0%, *P*=0.027). More than half of the women had received information from their GP, and this was similar for both groups (57.1 *vs* 54.0%, *P*=0.68). Very few women had received information from voluntary organisations. The organisation that was contacted for information most frequently was Breast Cancer Care; with women living in affluent areas were more likely to have received or remember having received information from them than women living in deprived areas (23.4 *vs* 11.0%, *P*=0.03).

There were differences between women living in affluent and deprived areas in terms of the types of media from which they had obtained information (Table 2

**Table 2:** Information-seeking behaviour of women from affluent and deprived areas from the media

**Information source**	**Affluent *n* (% of affluent. group) Total *n*=77**	**Deprived *n* (% of deprived group) Total *n*=100**	***χ*^2^ test results**
Magazines	39 (50.6%)	33 (33.0%)	*χ*^2^=5.6, DF=1
			*P*=0.02
Newspapers	35 (45.5%)	22 (22.0%)	*χ*^2^=10.96, DF=1
			*P*=0.0009
Books	20 (26.3%)	18 (18.0%)	*χ*^2^=1.76, DF=1
			*P*=0.18
Leaflets	38 (49.4%)	31 (31.0%)	*χ*^2^=6.16, DF=1
			*P*=0.013
TV news	35 (45.5%)	26 (26.0%)	*χ*^2^=7.29, DF=1
			*P*=0.007
TV documentaries	41 (53.2%)	39 (39.0%)	*χ*^2^=3.56, DF=1
			*P*=0.06
TV drama	13 (16.9%)	13 (13.0%)	*χ*^2^=0.52, DF=1
			*P*=0.47

See Table 1 legend.

). Women from affluent areas were more likely to have acquired information from magazines (50.6 *vs* 33.0%, *P*=0.02), from newspapers (45.5 *vs* 22.0%, *P*=0.0009) and from leaflets (49.4 *vs* 31.0%, *P*=0.013). Information was sought from books similarly in both groups (26.3 *vs* 18.0%, *P*=0.18). Women living in affluent areas were more likely to have obtained information from television news (45.5 *vs* 26.0%, *P*=0.007) than women from deprived areas. There was no statistical difference between the groups in terms of television documentaries (53.2 *vs* 39.0%, *P*=0.06) or television drama (16.9 *vs* 13.0%, *P*=0.47).

### SF 36

For each of the SF-36 scales, with the exception of bodily pain, a statistically significant difference was demonstrated between women living in affluent and deprived areas. Women living in deprived areas were more likely to have lower scores and thus better health status (Table 3

**Table 3:** SF 36 scale scores for women from affluent and deprived areas

**SF 36 scale**	**Affluent *n* Median, (IQR)**	**Deprived *n* Median, (IQR)**	**Mann–Whitney test**
Physical functioning	*n*=74	*n*=95	*Z*=−3.97
	80 (54–95)	61 (20–80)	*P*=0.0001
			
Role – physical	*n*=75	*n*=87	*Z*=−2.59
	100 (67–100)	50 (0–100)	*P*=0.0096
			
Bodily pain	*n*=75	*n*=100	*Z*=−1.65
	46 (44–100)	56 (44–100)	*P*=0.09
			
Mental health	*n*=75	*n*=96	*Z*=−3.36
	76 (64–88)	66 (45–84)	*P*=0.0008
			
Role –emotional	*n*=74	*n*=86	*Z*=−2.48
	100 (67–100)	100 (0–100)	*P*=0.01
			
Social functioning	*n*=76	*n*=99	*Z*=−2.04
	87 (78–100)	75 (50–100)	*P*=0.04
			
Vitality	*n*=75	*n*=96	*Z*=−2.39
	60 (45–80)	50 (30–65)	*P*=0.02
			
General health perception	*n*=74	*n*=88	*Z*=−3.09
	77 (52–87)	59 (35–77)	*P*=0.002

). The most significant differences were seen in the physical functioning, role – physical and mental health scales.

### Help-seeking behaviour

In order to discover the different ways women from different backgrounds respond to anxiety the following question was asked: *If you become anxious about your breast problem, which, if any, of the following are you most likely to do?* (Table 4

**Table 4:** Most likely action in response to anxiety of women from affluent and deprived areas

**Action if anxious about breast problem**	**Affluent *n* (% of affluent group)**	**Deprived *n* (% of deprived group)**	***χ*^2^ test results**
*Keep it to yourself*			
	*n*=67	*n*=74	
Yes	6 (9.0%)	13 (17.6%)	*χ*^2^=2.60
No	45 (67.2%)	42 (56.8%)	DF=2
Possibly	16 (23.9%)	19 (25.7%)	*P*=0.27
			
*Speak to family or friends*			
	*n*=65	*n*=73	
Yes	33 (50.8%)	39 (53.4%)	*χ*^2^=0.31
No	15 (23.1%)	14 (19.2%)	DF=2
Possibly	17 (26.2%)	20 (27.4%)	*P*=0.85
			
*Speak to your GP*			
	*n*=72	*n*=87	
Yes	51 (70.8%)	63 (72.4%)	*χ*^2^=2.73
No	13 (18.1%)	9 (10.3%)	DF=2
Possibly	8 (11.1%)	15 (17.2%)	*P*=0.25
			
*Contact breast care nurse*			
	*n*=68	*n*=70	
Yes	25 (36.8%)	26 (37.1%)	Fisher's exact test
No	28 (41.2%)	32 (45.7%)	*P*=0.74
Possibly	15 (22.1%)	12 (17.1%)	
			
*Contact hospital specialist*			
	*n*=71	*n*=79	
Yes	43 (60.6%)	53 (67.1%)	*χ*^2^=2.08
No	15 (21.1%)	18 (22.8%)	DF=2
Possibly	13 (18.3%)	8 (10.1%)	*P*=0.35
			
*Contact a voluntary organisation, such as BACUP or Tak Tent*			
	*n*=63	*n*=67	
Yes	3 (4.8%)	1 (1.5%)	Fisher's exact test
No	53 (84.1%)	55 (82.1%)	*P*=0.41
Possibly	7 (11.1%)	11 (16.4%)	

). There was no statistically significant difference between the groups for any of the options presented. The most likely action of the respondents if they became anxious was to speak to their GP (70.8% of respondents from affluent areas, 72.4% of respondents from deprived areas), contact hospital specialist (60.6 *vs* 67.1) or speak to family or friends (50.8 *vs* 53.4%).

### Anxiety-provoking issues

Data from responses to the question – *do you worry about any of the following?* are presented in Table 5

**Table 5:** Degree of anxiety in women from affluent and deprived areas

**‘Very much’ anxiety^a^**	**Affluent *n* (% of affluent group)**	**Deprived *n* (% of deprived group)**	***χ*^2^ test results**
Money	*n*=71	*n*=82	Fisher's exact test
	2 (2.8%)	10 (12.2%)	*P*=0.02
			
Job security	*n*=71	*n*=73	Fisher's exact test
	2 (2.8%)	2 (2.7%)	*P*=0.67
			
Breast cancer	*n*=74	*n*=93	*χ*^2^=1.06
	17 (23.0%)	28 (30.1%)	DF=1
			*P*=0.30
			
Other health problems	*n*=73	*n*=86	*χ*^2^=5.73
	6 (8.2%)	19 (22.1%)	DF=1
			*P*=0.02
Family problems	*n*=72	*n*=80	*χ*^2^=3.86
	5 (6.9%)	14 (17.5%)	DF=1
			*P*=0.049
			
Relationship problems	*n*=71	*n*=78	Fisher's exact test
	2 (2.8%)	5 (6.4%)	*P*=0.26

a This table demonstrates the respondents who responded the question; Do you worry about any of the following? by answering ‘very much’.

. The respondents were offered the option of *very much/somewhat/a little/not at all*. These data were analysed comparing the response ‘very much’ with all other responses. Only small numbers of women reported ‘very much’ anxiety, the commonest cause being anxiety about breast cancer, with no difference shown between women living in affluent and deprived areas (23.0 *vs* 30.1%, *P*=0.30). The areas which between group differences were detected all demonstrated greater anxiety in women living in deprived areas: anxiety regarding money (2.8 *vs* 12.2%, *P*=0.02), anxiety regarding other health problems (8.2 *vs* 22.1%, *P*=0.02) and anxiety about family problems (6.9 *vs* 17.5%, *P*=0.049).

## DISCUSSION

This paper describes the perceptions of women regarding care following diagnosis with breast cancer and their health status as measured by SF-36. It is clear that breast cancer casts a long shadow in terms of psychosocial impact. The data presented here have shed light on on-going anxiety for women with breast cancer several years beyond diagnosis and have demonstrated the need for continued access to personal and professional support.

Most women in the study obtained information from their hospital specialist. However, women from affluent areas were significantly more likely to do so compared with women from deprived areas. The data regarding information provided by this study related only to the sources of information rather than to the amount or quality of the information. Other research in the west of Scotland has indicated that the vast majority of cancer patients want to be informed about their illness. Meredith *et al* (1996) also found that more patients from affluent areas than from deprived areas wanted to know that their illness was cancer and wanted information about all possible treatments. Some years ago the GIVIO investigators (1986) in Italy found that the quality of information received by women with breast cancer was directly and independently related to length of education. There have been some studies which have concentrated on information sources. Ashbury *et al* (1998) asked a group of 913 cancer patients about helpful sources of information. Nurses (61%), specialists (61%) and other cancer patients (60%) were reported by respondents to be helpful sources of information. In the study by Meredith *et al* (1996) all patients reported a preference for the diagnosis to be given by a hospital specialist. One of the authors (Fallowfield *et al*, 1994, 1995) has previously reported 94% of patients in a sample of 101 expressing a desire for as much information as possible from their Oncologist.

It is not entirely clear why affluent women in this study were more likely than deprived women to report having received information from hospital specialists and breast care nurses. Several explanations are possible. One of the hospitals in the study did not appoint a breast care nurse until the latter part of the second year of the study (T Cooke, personal communication). The specialists in this particular hospital only saw women from deprived areas, so this may have biased the results. Although this may explain the predominance of affluent women having received information from a breast care nurse, it does not explain the difference with respect to breast specialists. However, specialists working in hospitals serving deprived communities may be more hard pressed and have less time to explore patients’ information needs, and less time to reinforce information regarding breast cancer. An alternative explanation is that more of the affluent women in this study remembered receiving information from hospital specialists and breast care nurses because it was their personal preference to seek information from these professionals, rather than any failure on the part of the health care professionals with respect to more deprived patients.

Few women in our study contacted voluntary organisations and there may be several reasons for this. CANCERBACUP set an office up in Glasgow after the period covered by this study. Their information was available at the time, but women may not recall the organisation that published the books they read some years earlier. Although we were aware that the answers to the questions posed would be subject to recall bias, we made the assumption that this would be the same for women from both affluent and deprived areas. For each of the possible media sources suggested (books, magazines, newspapers, TV) the affluent were more likely to have sought information. This may be because affluent women are more likely to buy publications in which articles about cancer occur. An information source missing from our questionnaire is the Internet. This would of necessity be included in any such questionnaire in the future. As our study population developed breast cancer in 1992 and 1993 this was not relevant as use of the Internet was minimal then.

This study is the first reported study to investigate the possible differences in information sources due to socioeconomic differences. It emphasises the important role which health professionals have as the source of information about breast cancer and its management. Further studies regarding information given to breast cancer patients need to explore whether there are different informational needs in affluent and deprived groups.

Professional support does not merely involve providing information. The long shadow cast by a cancer diagnosis includes dealing with symptoms that possibly indicate recurrence. When asked about their likely behaviour if they became anxious about breast cancer over 70% of women from both deprived and affluent areas reported they would contact their GP. This is perhaps not surprising in view of our previously reported finding of increased consultation rates following diagnosis (Macleod *et al*, 2000b).

The SF-36 questionnaire has previously been used in a number of general practice populations and people from deprived areas have had poorer scores (Brazier *et al*, 1992; Jenkinson *et al*, 1993; Hemingway *et al*, 1997) as demonstrated by the results discussed in this paper. But this study is the first in the UK to use SF-36 to study affluent and deprived populations with breast cancer. It is unclear why the result for bodily pain was an exception to the pattern seen in the other dimensions of the questionnaire and this finding requires further investigation. This was not found in a Californian study of breast cancer survivors (Ashing-Giwa *et al*, 1999). We have previously discussed the importance of comorbidity in understanding the known poorer outcome of deprived women with breast cancer (Macleod *et al*, 2000b). The results from this questionnaire demonstrate that deprived women are also more likely than affluent women to have psychological comorbidity some years after diagnosis. This emphasises the importance of the role of primary care in the on-going care of women with cancer (Campbell *et al*, 2002) and is particularly pertinent in light of previous research which has shown that increasing socioeconomic deprivation is associated with a higher prevalence of psychological distress and shorter consultations in general practice (Stirling *et al*, 2001).

Women living in deprived areas reported a greater degree of anxiety regarding money, other health problems and family problems. Pinder *et al* (1993) found clinical depression to be significantly more prevalent among women with advanced breast in the lower social classes. Similar results to those were obtained by Dean (1987) in a study of women with early breast cancer. They suggest that deprived women may experience increased ‘psychosocial adversity’ compared to more affluent women and may experience greater financial hardship. Our study supports these findings. All of these studies confirm the close interaction between physical and psychosocial issues. Although many authors accept the complexity of these issues, there is little literature on the particular psychosocial problems confronted by women of low socioeconomic status suffering from breast cancer. As Visser and Herbert (1999) concluded ‘more longitudinal studies are needed to unravel the role of psychosocial factors in cancer.’

It is clear that improving outcomes in terms of improving psychological and physical morbidity for deprived women with breast cancer is a complex matter. Health professionals, particularly in primary care, need to be alert to the greater psychological distress demonstrated by socioeconomically deprived women with breast cancer even some years after diagnosis. Further work is necessary to elicit the most appropriate ways of tackling these issues in primary care and to understand in greater detail the influence of psychosocial factors on experience and outcome.
